# Catastrophic Health Expenditures Across Insurance Types and Incomes Before and After the Patient Protection and Affordable Care Act

**DOI:** 10.1001/jamanetworkopen.2020.17696

**Published:** 2020-09-24

**Authors:** Charles Liu, Karan R. Chhabra, John W. Scott

**Affiliations:** 1 UCLA/VA National Clinician Scholars Program, University of California, Los Angeles; 2 Department of Surgery, Stanford University School of Medicine, Stanford, California; 3 Institute for Healthcare Policy and Innovation, University of Michigan, Ann Arbor; 4 Department of Surgery, Brigham and Women’s Hospital, Boston, Massachusetts; 5 Department of Surgery, University of Michigan School of Medicine, Ann Arbor

## Abstract

This cohort study analyzes changes in financial risk protection associated with implementation of the Patient Protection and Affordable Care Act (ACA) across income strata and insurance types.

## Introduction

One decade after passage of the Patient Protection and Affordable Care Act (ACA), despite substantial gains in insurance coverage, health care affordability remains a major concern among US residents.^1^ Premiums are increasingly unaffordable, and underinsurance—incomplete financial protection despite coverage—is increasingly common.^2^ Although previous research has shown that the ACA’s Medicaid expansions decreased out-of-pocket spending among low-income adults,^3^ broader trends in out-of-pocket spending have not been well characterized. We thus sought to analyze changes in financial risk protection associated with ACA implementation across all income strata and insurance types.

## Methods

We obtained income, insurance coverage, and spending data from a nationally representative sample of adults aged 20 to 64 years in the Medical Expenditure Panel Survey, collected from 2010 to 2017. Our primary outcome was catastrophic health expenditures, defined with the World Health Organization threshold of calendar-year out-of-pocket plus premium spending exceeding 40% of postsubsistence income^4^ (calendar-year income minus typical food and housing expenditures from the Bureau of Labor Statistics^5^). Interrupted time series analysis was used to evaluate changes in the rate of catastrophic expenditures, with an inflection point in January 2014, the start of full ACA implementation.^2^ Individuals were stratified for analysis by quartile of household income as a percentage of the federal poverty level and by insurance type (eFigure in the Supplement). We also analyzed individuals across insurance types within the lowest income quartile.

Analyses were performed with multivariable linear regression models adjusted for sociodemographic characteristics, self-reported health, and Census region (eTable in the Supplement). We adjusted for inflation using the Consumer Price Index.^6^ Cluster-robust standard errors and survey weights for national estimates were used, with a 2-tailed *P* value threshold of .05. Analysis was conducted with Stata/SE version 16.1.

This study followed the Strengthening the Reporting of Observational Studies in Epidemiology (STROBE) reporting guideline for cohort studies.

## Results

We identified 159 941 survey respondents (49.1% men; mean age, 41.8 years [SD, 12.6 years]), representing 186 million individuals annually after survey weighting. The number of uninsured nonelderly adults declined from 42.9 million (23.5%) in 2010 to 27.9 million (14.8%) in 2017, whereas those with Medicaid coverage increased from 11.0 million (6.0%) to 18.3 million (9.7%) (*P* < .001). Coverage gains were concentrated in the 2 lower income quartiles, in which the uninsured rate decreased from 44.1% to 28.6% (lowest quartile) and 27.0% to 18.7% (*P* < .001).

**Figure 1.**
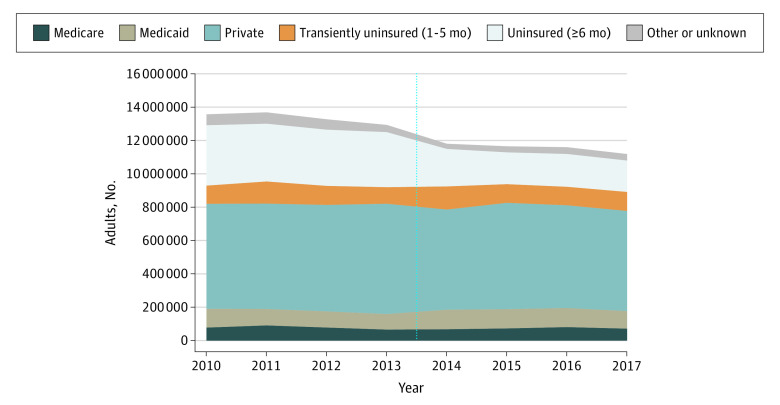
Number of Adults Aged 20 to 64 Years Experiencing Catastrophic Health Expenditures, by Insurance Type The total sample included 159 941 patients, and the weighted sample included 186 048 287 per year. Insurance type was defined as uninsured (≥6 months), transiently uninsured (1-5 months), or, given year-round coverage, as the insurer with the greatest share of calendar-year health care expenditures. Other or unknown includes individuals with year-round Veterans Affairs or Tricare coverage, or year-round insurance coverage from an unknown source. The vertical line indicates the date of Patient Protection and Affordable Care Act implementation.

The number of adults experiencing catastrophic expenditures yearly declined from 13.6 million (7.4%) in 2010 to 11.2 million (5.9%) in 2017 (*P* < .001) (Figure 1). Privately insured adults composed 46.4% of catastrophic expenditure cases in 2010 and 53.6% in 2017 (*P* < .001).

**Figure 2.**
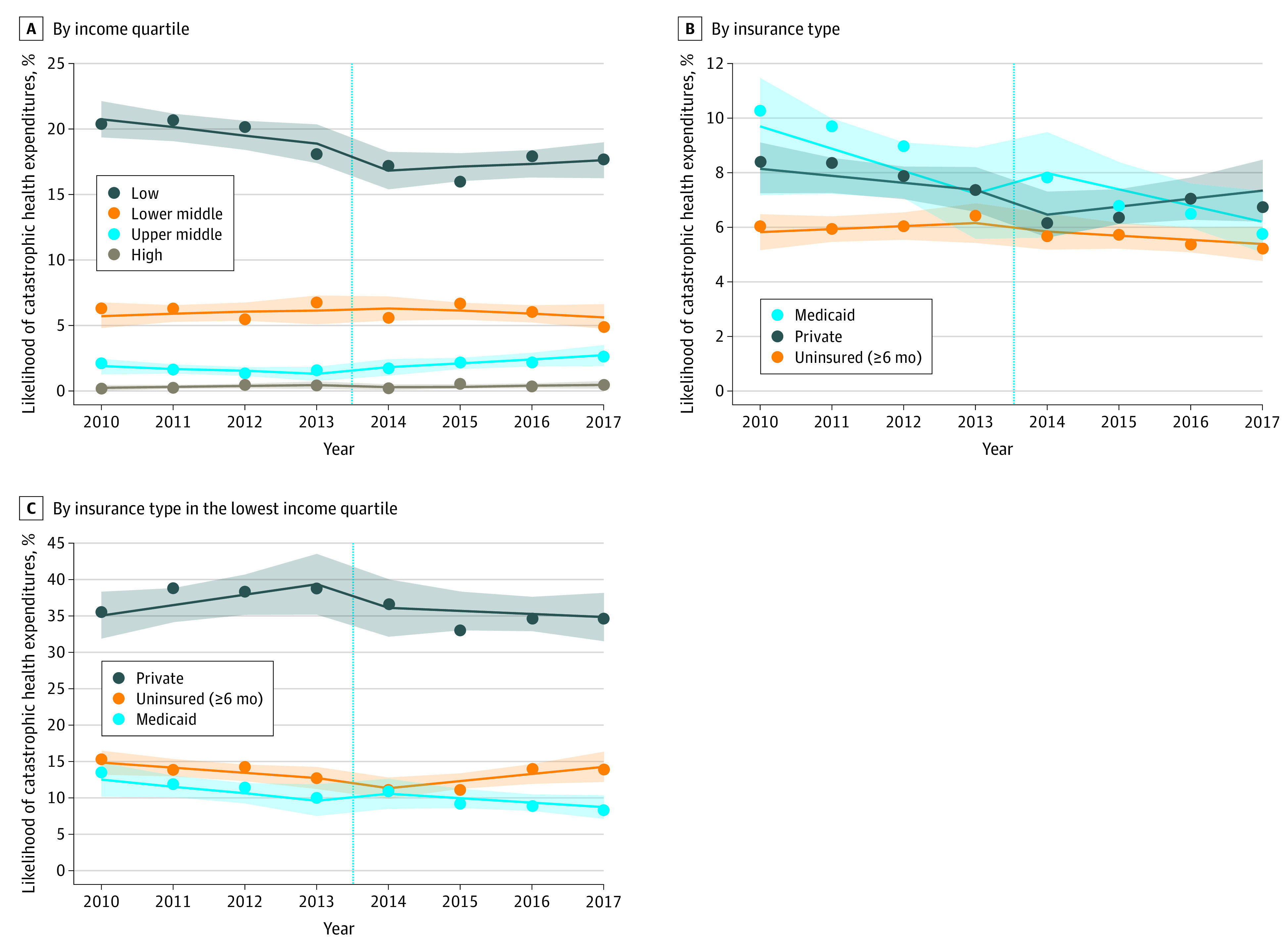
Changes in Likelihood of Catastrophic Health Expenditures Among Adults Aged 20 to 64 Years, by Income Quartile, Insurance Type, and Insurance Type Within the Lowest Income Quartile For clarity, only the 3 most common insurance types are shown. A and B, The sample included 159 941 patients, and the weighted sample included 186 048 287 per year. C, The sample included 57 224 patients, and the weighted sample included 46 518 845 per year. Markers indicate mean likelihood; lines, best fit line; shaded areas, 95% CIs; and vertical line, Patient Protection and Affordable Care Act implementation.

In our interrupted time series analysis, individuals in the lowest income quartile experienced a 2.3 percentage point decrease in likelihood of catastrophic expenditures (95% CI, −4.6 to −0.1) (Figure 2A), whereas no change was observed in other income quartiles. Stratified by insurance type, privately insured individuals experienced no change in catastrophic expenditures (adjusted change, −0.2 percentage point; 95% CI, −1.4 to 1.0) (Figure 2B). Finally, in our subanalysis of the lowest income quartile, privately insured individuals again experienced no change (adjusted change, −2.8 percentage points; 95% CI, −9.5 to 3.8) (Figure 2C), and in fact had the highest rate of catastrophic spending in 2017 (34.6% vs 8.3% among Medicaid enrollees and 13.9% among the uninsured).

## Discussion

ACA implementation was associated with 2 million fewer US adults with catastrophic expenditures each year. Financial protection improved for the lowest income quartile, which was one of the ACA’s principal aims. However, improvements were not observed in higher income quartiles or among the privately insured, who represent an increasing share of those experiencing catastrophic expenditures. Among individuals in the poorest quartile, the privately insured are the most vulnerable, with one-third experiencing catastrophic spending annually. These findings help to explain why so many US residents, including those with insurance, continue to worry about their ability to afford needed care.

Limitations include changing patient composition within insurance groups, meaning our analysis evaluates financial protection currently conferred by each insurance type, rather than the effect of gaining coverage. Also, because the Medical Expenditure Panel Survey does not quantify unpaid bills or medical debt, our analysis likely underestimates patients’ true financial hardship. Last, changes in catastrophic spending could have gone undetected in subgroups with small sample size, such as low-income privately insured individuals.

Despite large coverage gains, 11 million US adults, including 6 million with private insurance, continue to experience catastrophic health expenditures annually. These figures are likely to increase as millions lose employment or require unexpected medical care because of coronavirus disease 2019. Health reform should move beyond expanding insurance coverage alone to address persistently high out-of-pocket spending among the insured.

## Notes

Supplement. **eFigure. **Flow diagram for determination of insurance type **eTable.** Covariates used for adjustment in interrupted time series analysis
