# Supplementary material for: Catastrophic Health Expenditures Across Insurance Types and Incomes Before and After the Patient Protection and Affordable Care Act
Source: JAMA Netw Open. 2020 Sep 24;3(9):e2017696. doi: 10.1001/jamanetworkopen.2020.17696 (PMC7516626; doi:10.1001/jamanetworkopen.2020.17696)
Supplement: Supplement. — eFigure. Flow diagram for determination of insurance type eTable. Covariates used for adjustment in interrupted time series analysis [file jamanetwopen-e2017696-s001.pdf]

Supplementary Online Content

Liu C, Chhabra KR, Scott JW. Catastrophic health expenditures across insurance types and incomes before and after the Patient Protection and Affordable Care Act. *JAMA Netw Open*. 2020;3(9):e2017696. doi:10.1001/jamanetworkopen.2020.17696

eFigure. Flow diagram for determination of insurance type

eTable. Covariates used for adjustment in interrupted time series analysis

This supplementary material has been provided by the authors to give readers additional information about their work.

© 2020 Liu et al. *JAMA Network Open.*

eFigure. Flow diagram for determination of insurance type

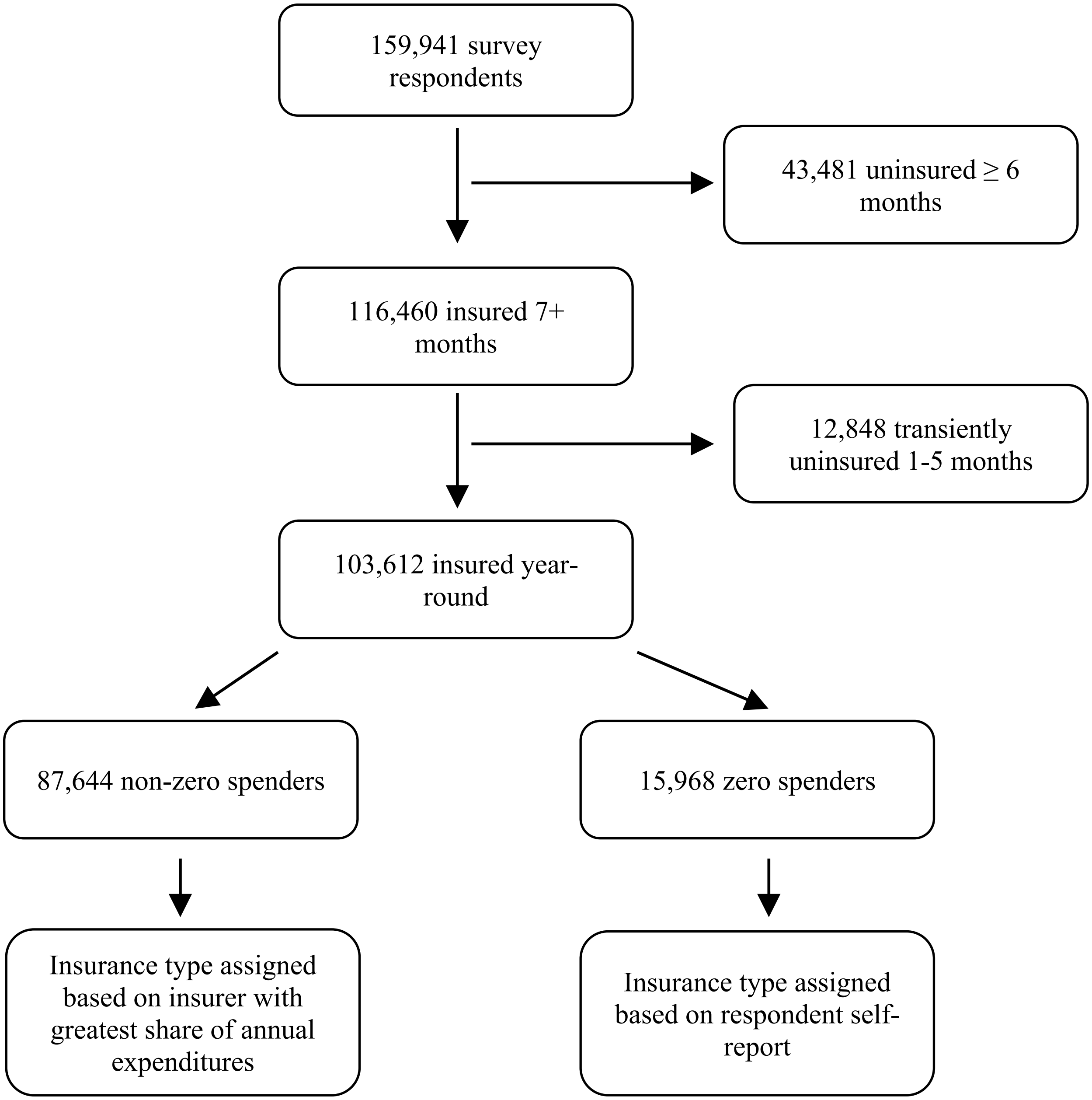

Notes: Here, zero vs non-zero spending refers to total expenditures for healthcare utilization (excluding premiums), including patient out-of-pocket spending and spending by insurers. Zero spenders could still experience catastrophic expenditures if their premiums alone exceeded catastrophic thresholds.

We used self-reported coverage for zero-spenders, with Medicare assumed to be the primary insurer when present, followed by Medicaid, then Tricare, then private insurance, then other/unknown (including VA and Tricare).

The final 6 possible insurance types were: uninsured (≥ 6 months), transiently uninsured (1-5 months), Medicare, Medicaid, private insurance, and other/unknown.

© 2020 Liu et al. *JAMA Network Open.*

eTable. Covariates used for adjustment in interrupted time series analysis

**Gender**
Male
Female
**Age**
20-25
26-35
36-45
46-55
56-64
**Race/Ethnicity**
Non-Hispanic White
Non-Hispanic Black
Hispanic
Asian
Other or Multiple
**Marital Status**
Married
Widowed/Divorced/Separated
Never Married
Unknown
**Employment Status**
Unemployed
Employed
**Family Size (categorical, 1-16)**
**Self-Reported Health Status**
Excellent/Very Good/Good
Fair/Poor
Unknown
**Census Region**
Northeast
Midwest
South
West

© 2020 Liu et al. *JAMA Network Open.*
